# Disparity and Convergence: Chinese Provincial Government Health Expenditures

**DOI:** 10.1371/journal.pone.0071474

**Published:** 2013-08-19

**Authors:** Jay Pan, Peng Wang, Xuezheng Qin, Shufang Zhang

**Affiliations:** 1 West China School of Public Health, Sichuan University, Chengdu, China; 2 Western China Center for Rural Health Research and Development, Sichuan University, Chengdu, China; 3 School of Economics, Southwest University for Nationalities, Chengdu, China; 4 School of Economics, Peking University, Beijing, China; 5 United Nations Research Institute for Social Development, Geneva, Switzerland; Universidad Veracruzana, Mexico

## Abstract

The huge regional disparity in government health expenditures (GHE) is a major policy concern in China. This paper addresses whether provincial GHE converges in China from 1997 to 2009 using the economic convergence framework based on neoclassical economic growth theory. Our empirical investigation provides compelling evidence of long-term convergence in provincial GHE within China, but not in short-term. Policy implications of these empirical results are discussed.

## Introduction

Health is human beings' basic need and belongs to human beings' “substantial freedom” [1]. As merit goods, health has strong positive externalities and plays an important role in promoting social and economic development [2], [3], [4]. Thus, improving the population health is one of the most important social objectives of most governments around the world today. A common approach to achieve this goal is to set up a health system to guarantee that all residents suffering from illness can enjoy the basic medical services, and to guarantee the equity in the distribution of health [5].

To that purpose, although more expenditure on health does not guarantee better health outcome, governments usually focus on the operational measure of promoting equality of government health expenditure (GHE) on a sub-country level. Numerous empirical studies have shown that there are great within-country regional disparities in GHE for both developed and developing countries [6], [7], [8]. China is no exception. In 1997, GHE per capita in Shanghai (185 Yuan) was the highest among all provinces and is 9 times greater than the lowest Shandong Province's expenditure (18 Yuan), after adjusted using the consumer price index (CPI) with 2009 = 100. In 2000, WHO ranked China 188^th^ out of the 191 Member States in terms of fairness of financial contribution to health systems [9].

Besides the impact of different economic development level across provinces, China's 1994 fiscal reform, which stipulates a rearrangement of the tax structure, also contributes to the huge regional disparity in GHE. The new Tax-Sharing System (TSS) reduced the local governments' tax revenues, but their responsibilities of providing public services remained unchanged. In addition, TSS introduced a highly disequalizing feature to revenue sharing by requiring the central government to share value-added tax (VAT) revenues with local governments at a flat rate by origin, ensuring that revenue-rich regions keep more. For a detailed discussion of the effect of China's 1994 fiscal reform, please refer to reference [9]. Governments of poorer provinces constrained by their budget could invest only very limited amount in health sector.

Another source of the regional disparity in GHE lies in the structure of healthcare financing system. Before the market reform starting from the late 1970 s, health insurance schemes were organized around the workplace. The Cooperative Medical Scheme (CMS) financed healthcare for members of the agricultural commune, whereas the Labor Insurance Scheme (LIS) and Government Insurance Scheme (GIS) financed healthcare for state-owned enterprise (SOE) workers and government officials, respectively. Most urban and rural residents were covered by one of these schemes. The market-liberalization process broke the foundation for CMS and LIS, forced both of them to collapse in many regions. As a result, insurance coverage among both urban and rural residents plummeted (e.g., [10]). From late 1970 s to 2003, almost all GHE was used to subsidize the public hospitals. Under the supply-side financing, the government subsidies to public hospitals are based on their scales, i.e., large tertiary hospitals get most subsidies and smaller-scale township hospitals get little. Since bigger hospitals are more concentrated in the economically developed provinces, supply-side financing does little to help to reduce the regional disparities in GHE, and may even contribute to the widening GHE gap.

Although there is a huge disparity in GHE in China, we expect a convergence in GHE across provinces in recent years. From a general economic perspective, the production function of GHE should exhibit diminishing marginal returns. Thus, provincial governments with higher levels of GHE have fewer incentives to expand spending in health sector than those provincial governments with relatively lower GHE. If this is the case, there might be a catching up of provinces with lagged-behind GHE. However, whether provincial GHE converges depends on the income elasticity of the GHE. Previous studies (e.g., [11], [12]) find an income elasticity of health expenditures to be equal or greater than 1. If this holds, GHE convergence remains a question to be tested by empirical study.

The demand-side financing that subsidizes the large-scale public health insurance system was introduced by the Chinese government in recent 10 years. New Rural Cooperative Medical Scheme (NRCMS) was established in 2003, and continued with Urban Resident Basic Medical Insurance (URBMI) in 2007. The government financing of healthcare changed from a dominant supply-side subsidy to a more balanced mixture of demand- and supply-side subsidies. (Before 2003, there were two government-run or government-regulated medical insurance schemes in China: Government Employee Medical Insurance (GEMI) and Urban Employment Basic Medical Insurance (UEBMI). Government was responsible to recover healthcare costs of the GEMI beneficiaries. Thus even before 2003, demand-side government financing of healthcare existed but the scale was very small compared with supply-side financing. After 2003, the newly established NRCMS targeting at rural population has been heavily financed by fiscal expenditures. URBMI, which targets to provide insurance protection for children, the elderly, the disabled, and other non-working urban residents, is also subsidized by government. On the contrary, UEBMI which covers urban people employed in formal sectors is mostly financed by payments from employers and individuals. Due to the broad and increasing coverage of NRCMS and URBMI, GHE going to the demand-side in the form of insurance premium subsidy has increased substantially.) Under the new framework, GHE should be proportional to the number of insurance enrollees in the province, which is highly correlated with the total population size. Thus, the per capita GHE should exhibit a converging trend across provinces when the government healthcare subsidy shifts towards the demand-side. In 2009, China launched a new round of health reforms in which the government planned to invest 850 billion Yuan ($124 billion) in the health sector during the subsequent 3 years [13]. Two thirds of this investment would be spent to subsidize the demand-side. A quick convergence in provincial GHE was expected.

Although there are some studies examining the regional disparity of GHE in China (e.g., [14]) and the time trend of disparity in total health expenditures (THE), few studies, to our knowledge, has systematically examined the time trend of the GHE disparity across different provinces in China. For example, Chou and Wang (2009) examines the convergence of THE from 1978 to 2004 in China, but their study doesn't distinguish government health expenditures with private health expenditures, thus has very different policy implication from this paper [15]. Moreover, the existing studies are based on data prior to 2006, thus they are unable to examine the effects of introducing demand-side financing on GHE equality. As the national development guideline (12^th^ 5-Year Plan) made it clear that the new health reforms aim to provide basic healthcare as public goods and to promote equalization of public services, it is crucial to understand the right policy mix to achieve equitable distribution of government health resources. This paper addresses regional inequality in GHE and provides empirical evidence on whether provincial GHE converges in China from 1997 to 2009 in both the long and short term. An understanding of this issue can be useful in evaluating health policies recently introduced by the central and local governments.

The paper proceeds as follows: Section 2 introduces the analytical framework, the data source, and the variable specifications; Section 3 presents empirical results; Section 4 discusses the significance and limitation of our results; and Section 5 concludes the paper and offers policy implications.

## Data and methods

### Data

We used a sample of panel data covering 31 provinces in People's Republic of China during 1997–2009. 31 Provinces refer to 22 provinces (Hebei, Shanxi, Liaoning, Jilin, Heilongjiang, Jiangsu, Zhejiang, Anhui, Fujian, Jiangxi, Shandong, Henan, Hubei, Hunan, Guangdong, Hainan, Sichuan, Guizhou, Yunnan, Shaanxi, Gansu, Qinghai,), 4 municipalities (Beijing, Tianjin, Chongqing, Shanghai) and 5 autonomous regions (Inner Mongolia, Guangxi, Ningxia, Xinjiang, Tibet). Taiwan province and two special administrative regions, Hong Kong, and Macao are excluded from the sample. These data were compiled from annual publication of Yearbook of Public Health in Peoples' Republic of China (1998–2010) and China Statistical Yearbook (1998–2010). GHE and GDP were converted into real per capita form by dividing by the provincial population and deflating using the consumer price index (CPI) with 2009 as base year. The CPI information is directly obtained from China Statistical Yearbook (1998–2010) and population densities are deduced from provincial population data from each year's China Statistical Yearbook and provincial land size data from China 2000 Census.

GHE, social health expenditures (SHE), and residents' health expenditure (RHE) constitute the total health expenditure (THE) [8]. Thus, THE is the sum of government health expenditure, and the social organizations' and residents' expenditure on health in a given year. In 2009, the shares of GHE, SHE and RHE in THE are 27.2%, 34.6%, and 38.2%, respectively, which shows GHE is an important component of THE. According to the Yearbook of Public Health in Peoples' Republic of China (2010), the GHE proportion in THE was 16.4% in 1997, and fluctuated during 1997–2002, then kept increasing to reach 27.2% in 2009. In general, GHE includes the health expenditure at all government levels, which could be divided into two parts in China, one is central GHE, and the other is local (provincial) GHE. Since this study aims to examine the regional disparity in GHE, we focus on the latter one which accounts for 98.4% of total GHE in 2009. Basing on Pan and Liu [8], Chinese GHE includes the following 10 components: health recurrent budget, family planning budget, health administration budget, Chinese medical budget, food and drug supervision and administration budget, health research funding, infrastructure budget, health administration and insurance management fees, other government health departments budget, and basic health insurance fund subsidies.

### Methodology and empirical strategy

As the Chinese government holds the policy goal to narrow regional disparity in economic and social development, we can expect a converging trend of provincial GHE if the aforementioned policy interventions of large-scale social insurance are effective. The neoclassical growth model analyzed two different types of convergence, σ-convergence and β-convergence. Both of them are widely applied in empirical studies on the time trend of regional disparities, including the analysis of health expenditure [16], [17]. The two concepts are also employed in our empirical examination.

The concept of σ-convergence introduced by Barro and Sala-i-Martin [18] refers to the scene that the dispersion of income per capita across countries displays a tendency to decline over time. Two popular measures of dispersion used in the literature are the coefficient of variation (CV), and the standard deviation of log income per capita (SD) [19]. In our study, σ-convergence refers to the status that the degree of variation of provincial GHE per capita decreases with time in a given period. Consistent with previous research in this context, CV and SD are used to measure the extent of the convergence process across provinces:
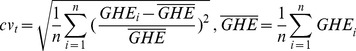
(1)




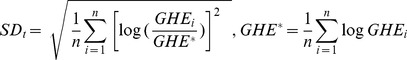
(2)


The concept of β-convergence implies that poor countries grow faster than rich countries, and is typically tested by running the “Barro regression” [20], which involves regressing the growth rate in GDP per capita on its initial level for a given group of countries [21]. In this paper, β-convergence refers to the situation that the growth rate of GHE per capita is higher in regions with lower initial level than in regions with higher initial values. We examine the existence of β-convergence using the following regression model:
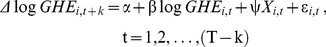
(3)Where *ΔlogGHE_i,t+k_* is the difference between *logGHE_i,t+k_* and *logGHE_i,t_*, *X_i,t_* is the vector of other independent variables that includes GDP per capita, the population density, the ratio of female in the total population, and the ratio of inactive in the total population. Non-labor force people are defined as people under 15 or above 64. By definition, if β-convergence exists, we should have *β*<0. When *X_i,t_* is not included, the estimated value of *β* reflects absolute convergence. When the control variable vector *X_i,t_* is added to the model, the estimated value of *β* reflects conditional convergence, i.e. the velocity of convergence is conditional on the other factors.

It should be pointed out that the subscript *t* indicates the time interval. The estimation of *β* implies the growth rate in GHE per capita over the *t*-year period. Thus, the different time-span estimations of β-convergence are based on the specific time interval setting. Following the literature [15], [16], the short and long term are defined as one year and twelve years span in this study, respectively.

## Results

### σ-convergence

**Figure 1:**
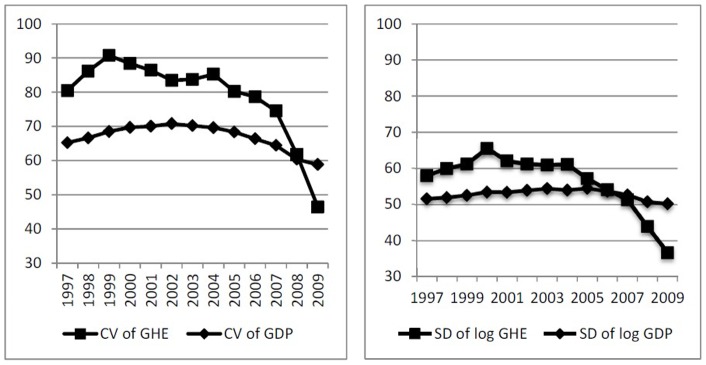
σ-convergence of GHE per capita in China (1997–2009).

We first examine the σ-convergence of GHE per capita using CV and SD. Figure 1 depicts the dynamic change in CV and SD of GHE per capita among the 31 provinces in China from 1997 to 2009. (Due to space limit, we do not report the mean, CV, and SD of GHE per capita here. Interested readers can contact with the author for these results.) For comparison, we also add the curve showing the changing dynamics of CV and SD of GDP per capita in the same time period. In short, the CV and SD of GHE demonstrate a similar converging trend. The cross-provincial CV and SD of GHE per capita had been rising before year 2004, but had decreased dramatically after 2004, especially during 2007 to 2009. By contrast, the cross-provincial CV and SD of GDP per capita had stayed relatively flat during the same period. Although regional disparity of GDP per capita also displays a deceasing trend after 2004, the reduction rate of GHE disparity is much higher. In year 2004, the CV and SD of GHE per capita were 22.47% and 12.50% higher than that of GDP per capita; however, they became 21.08% and 26.96%, respectively, lower than that of GDP per capita in 2009.

In order to statistically test for σ-convergence, a simple F-test (one-sided) proposed by Nixon [16] was applied. The null hypothesis (σ^2^_1997_ ≤σ^2^_2009_) for CV and SD were rejected at the 1% level. This suggests that the cross-provincial GHE per capita demonstrated σ-convergence after 2004, and converges faster than GDP per capita. We also carried out more statistical tests, Table 1 shows the results.

**Table 1:** σ-convergence and F-test.

Year	CV of GHE	F-test	P-value	SD of log GHE	F-test	P-value
1997	80.483	–	–	58.043	–	–
1998	86.188	0.872	0.645	59.984	0.936	0.571
1999	90.739	0.787	0.786	61.248	0.898	0.615
2000	88.422	0.828	0.696	65.550	0.784	0.745
2001	86.444	0.867	0.652	62.080	0.874	0.643
2002	83.484	0.929	0.579	61.235	0.898	0.615
2003	83.741	0.924	0.586	60.957	0.907	0.606
2004	85.269	0.891	0.624	61.120	0.902	0.611
2005	80.243	1.006	0.495	57.207	1.029	0.469
2006	78.669	1.047	0.451	54.137	1.150	0.353
2007	74.497	1.167	0.338	51.308	1.280	0.252
2008	61.738*	1.699	0.076	43.897*	1.748	0.066
2009	46.430***	3.005	0.002	36.687***	2.503	0.007

Note: F-test for CV,  =  CV_1997_^2^/CV_t_^2^, where1997 is the base year, and t is the year under test. The null hypothesis is CV^2^_1997_ ≤CV^2^_t_. The F-test for SD is by the same token. * significant at 10%, *** significant at 1%.

### β-convergence

**Figure 2:**
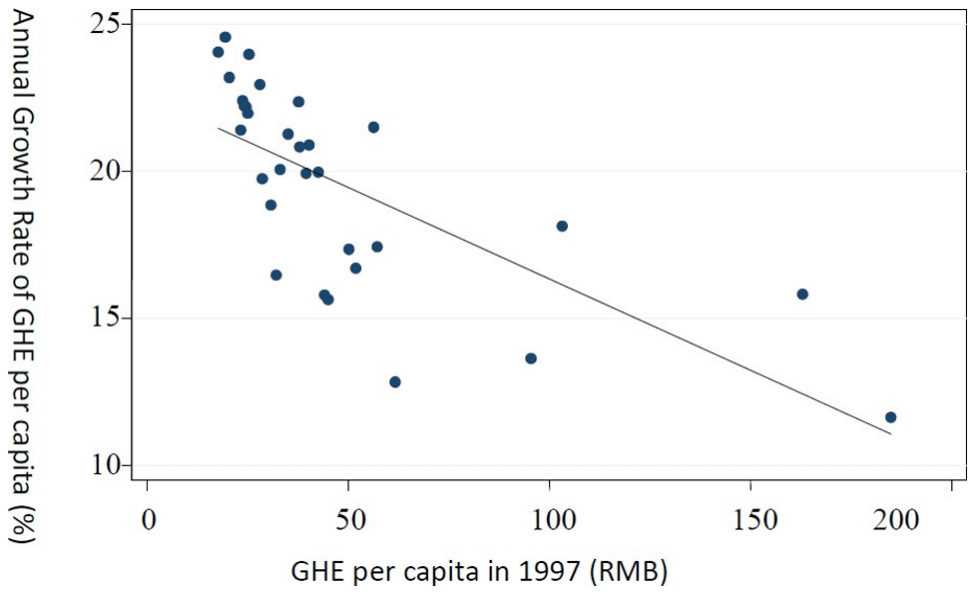
GHE per capita in 1997 and annual growth rate of GHE per capita from 1997–2009.

Figure 2 shows the relationship of the initial level of GHE per capita in 1997 and its average growth rate during the 1997–2009 period, which indicates a strong negative correlation and provides preliminary support for β-convergence.

We then formally test the existence of β-convergence using the regression approach. The results based on equation (3) are presented in Table 2, with Model (1) and (2) testing the short-term β-convergence (k = 1), and Model (3) and (4) testing the long-term β-convergence (k = 12). Model (1) examines the short-term absolute β-convergence. The coefficient estimate of previous year's GHE per capita is positive and significant at the 1% level. The hypothesis of absolute β-convergence is thus rejected. The result indicates that the regional disparity of GHE per capita widens at an annual rate of 5.57% in the unconditional term. Under the framework of conditional convergence, we add GDP per capita, the population density, the ratio of females in the total population, and the ratio of non-labor force in the total population in Model (2) to control for provincial economic and demographic characteristics. Under this specification, the coefficient estimate of previous year's GHE per capita becomes insignificant, but is still positive. This suggests that the regional disparity of GHE per capita is not widening conditional on the economic and demographic trend, but also no convergence can be observed for the short term.

**Table 2:** Regression Results for β-convergence.

	(1)	(2)	(3)	(4)
Log GHE	0.0557***	0.0210	−0.4813***	−0.1985*
	(0.0139)	(0.0238)	(0.0669)	(0.1135)
Log GDP		0.1159***		−0.5078***
		(0.0363)		(0.1261)
Population density		−0.0001***		0.0000
		(0.0000)		(0.0001)
Ratio of Female		−1.8856*		−3.3135
		(1.0731)		(6.3611)
Ratio of Non-labor force		0.2205		−4.2250***
		(0.3527)		(1.0082)
Constant	−0.0631	−0.0925	3.9095***	10.3288***
	(0.0619)	(0.6593)	(0.2321)	(3.4678)
Observations	372	372	31	31
R-squared	0.0620	0.1427	0.6397	0.8413

Note: Each column represents a regression basing on equation (3). Column (1) and (2) tests the short-term β-convergence (k = 1), and Column (3) and (4) tests the long-term β-convergence (k = 12). Robust standard errors in parentheses, * significant at 10%, *** significant at 1%.

Model (3) and (4) examine the long-term β-convergence when k = 12. Contrary to the short-term effect, these two models both have negative and statistically significant coefficient estimates for initial level of GHE per capita. This finding indicates that provinces with lower initial level of GHE per capita enjoy greater growth rate under both conditional and unconditional framework. The absolute and conditional (with economic and demographic characteristics controlled) β-convergence are supported, and this result is also consistent with Figure 2.

Besides looking into all provinces in China, we also examine whether the GHE convergence occurs within a region. To take the geo-economic factors into account, we followed the “Convergence Club” concept by Cai et. al. [22] and categorized the 31 provinces into 3 regional sub-samples (East, Middle, and West) in accordance with the standards of the National Bureau of Statistics of China. We examine short-term unconditional and conditional β-convergence (k = 1) within each region. Because of the sample size limit (there are only 12, 9, and 10 provinces in the East, Middle, and West sub-samples respectively), we don't examine the sub-sample long-term β-convergence (k = 12). As indicated in Table 3, the results suggest that β-convergence is not supported within each of the sub-samples.

**Table 3:** Regression Results for Convergence Club β-convergence.

	East		Middle		West	
	(1)	(2)	(3)	(4)	(5)	(6)
GHE	0.0387***	0.0122	0.1657***	0.0137	0.0451	−0.0824
	(0.0111)	(0.0404)	(0.0159)	(0.0498)	(0.0384)	(0.0939)
Control variables	No	Yes	No	Yes	No	Yes
N	144	144	108	108	120	120
R-squared	0.0665	0.2167	0.4683	0.6265	0.0219	0.1416

Note: Each column represents a regression to test the short-term β-convergence (k = 1) within a regional (east, middle, and west) sub-sample. Control variables include GDP per capita, the population density, the ratio of females in the total population, the portion of adopted population in the total population. Robust standard errors in parentheses, *** significant at 1%.

## Discussion

Our findings of the σ-convergence during 2004–2009 suggest that the Chinese central government's efforts to promote the cross-regional equalization of GHE are effective. Since the inception of NRCMS and URBMI in 2003 and 2007 respectively, the coverage rate and financing level for the two insurance programs have grown rapidly. For example, until 2009, the government subsidy for NRCMS premium reached 80 Yuan per insured person, accounting for 22% of the average provincial GHE per capital, and the national coverage rate reached 94%. The expansion in enrollment and the government subsidy are expected to work together to boost the overall GHE level and to promote its equality across different regions. Accordingly, our empirical result shows σ-convergence of the provincial GHE starting in 2004 and further accelerating since 2007 which coincided with the switch of government's massive investment in social health insurance programs. Our findings provide indirect evidence in support of the hypothesis that changing the subsidization strategy from a supply-side dominant to a more balanced approach of demand- and supply-side subsidy could lead to a convergent trend of the cross-regional GHE in China.

However, our results on the β-convergence caution us that China's achievement in promoting health equality may not be as significant as suggested by the descriptive analysis. The significantly positive coefficient of lag GHE in Model (1) indicates that, contrary to what the absolute β-convergence refers to, the provinces with lower initial GHE level actually experienced lower GHE growth rates than provinces with higher initial GHE level. The regional disparity of GHE growth rate hence widens. Since GHE per capita is positively correlated with economic prosperity represented by GDP per capita, this result might be caused by the fact that richer regions incline to allocate more public resources in promoting population health. The literature widely documents that health expenditure is indeed more of a luxury good than a necessity [23], [24]. The conditional framework supports our conjecture that the coefficient of GDP is positive and statistically significant at the 1% level, but we do not find evidence supporting the existence of β-convergence in the short term (one year span). However, the coefficient of lag GHE becomes insignificant which suggests that the regional disparity of GHE per capita is not widening conditional on the economic and demographic trend, but also no convergence is observed in the provincial GHE for the short run. The results of short-term and long-term (twelve years span) β-convergence seem to be conflicting. However, the findings are reasonable if we take into account that in the short run, local governments cannot be flexible enough to adjust their expenditures in health because they are limited by the inertia of the budget and fiscal mechanisms. However, in the long run, the regional GHE can be adjusted and to a new equilibrium level according to local demand and central policies.

Recalling that the ultimate goal of promoting GHE equity lies in promoting health equity, we also test the β-convergence of two extremely relevant variables: provincial resident health expenditure (RHE) and provincial mortality rates. RHE represents the non-government expenditure on health, and provincial mortality rates represent the population health. Regression results are provided in Table 4. Significant absolute and conditional β-convergence of both RHE and mortality rates are found in the short and long term. Although our study does not provide direct evidence on the causal effect of GHE and RHE convergence on mortality convergence, we may infer from our estimation that there might be a positive impact of GHE and RHE convergence on population health equalization for long term.

**Table 4:** Regression Results of RHE and Mortality for β-convergence.

	(1)	(2)	(3)	(4)
Log RHE	−0.3275***	−0.3270***		
	(0.0521)	(0.0494)		
Mortality			−0.0189***	−0.0255***
			(0.0043)	(0.0053)
Log GDP		−0.0117		−0.0218**
		(0.1202)		(0.0089)
Population density		0.0001		0.0000
		(0.0001)		(0.0000)
Female		36.5765***		0.3369
		(8.2240)		(0.4163)
Adopted		2.8844		−0.0999
		(1.9546)		(0.1208)
Constant	2.0248***	−16.6962***	1.1094***	1.2132***
	(0.3016)	(4.1159)	(0.0264)	(0.2190)
Observations	370	370	369	369
R-squared	0.1971	0.2600	0.0586	0.0791

Note: Each column represents a regression. Column (1) and (2) test the short-term (k = 1) β-convergence in RHE, and Column (3) and (4) test the short-term (k = 1) β-convergence in Mortality. Robust standard errors in parentheses, ** significant at 5%, *** significant at 1%.

Moreover, if GHE could promote population health, we would consider the incremental changes of population health as GHE's marginal products. Given the assumption of decreasing marginal products of GHE, the optimal health outcome would be achieved by equalization of GHE in all regions if the initial population health among regions is the same. Furthermore, considering different initial health levels, the optimal GHE per capita in different regions should be negatively correlated with the initial regional population health level, so that equal marginal benefits across different regions can be achieved.

## Conclusion

This paper applies the concept of convergence from the neoclassical economic growth theory to test whether the Chinese provincial GHE converge during 1997 to 2009. Using province level panel data, our main findings are: (1) The variation of GHE per capita has decreased since 2004, thus provides support for σ-convergence, and the convergence of GHE per capita outpaced that of GDP per capita. (2) Provincial GHE per capita demonstrates neither absolute β-convergence nor conditional β-convergence in the short term. However, both absolute β-convergence and conditional β-convergence are supported in the long term, indicating long-term “Catching-up” effect. (3) Results based on Convergence Clubs (East, Middle, and West) show no evidence of short-term β-convergence at the regional level, either.

We nevertheless recognize that the validity of the results and the corresponding policy implications may suffer from the effects of unobserved determinants of health and from simultaneity problems. A further study with a longer time series of smaller areas can mitigate some of these limitations.
